# Supplementary material for: Long noncoding RNA licensing of obesity-linked hepatic lipogenesis and NAFLD pathogenesis
Source: Nat Commun. 2018 Jul 30;9:2986. doi: 10.1038/s41467-018-05383-2 (PMC6065308; doi:10.1038/s41467-018-05383-2)
Supplement: Supplementary file 1 — Supplementary information [file 41467_2018_5383_MOESM1_ESM.pdf]

SUPPLEMENTARY INFORMATION

Long noncoding RNA licensing of obesity linked hepatic lipogenesis and NAFLD pathogenesis

Zhao et al.

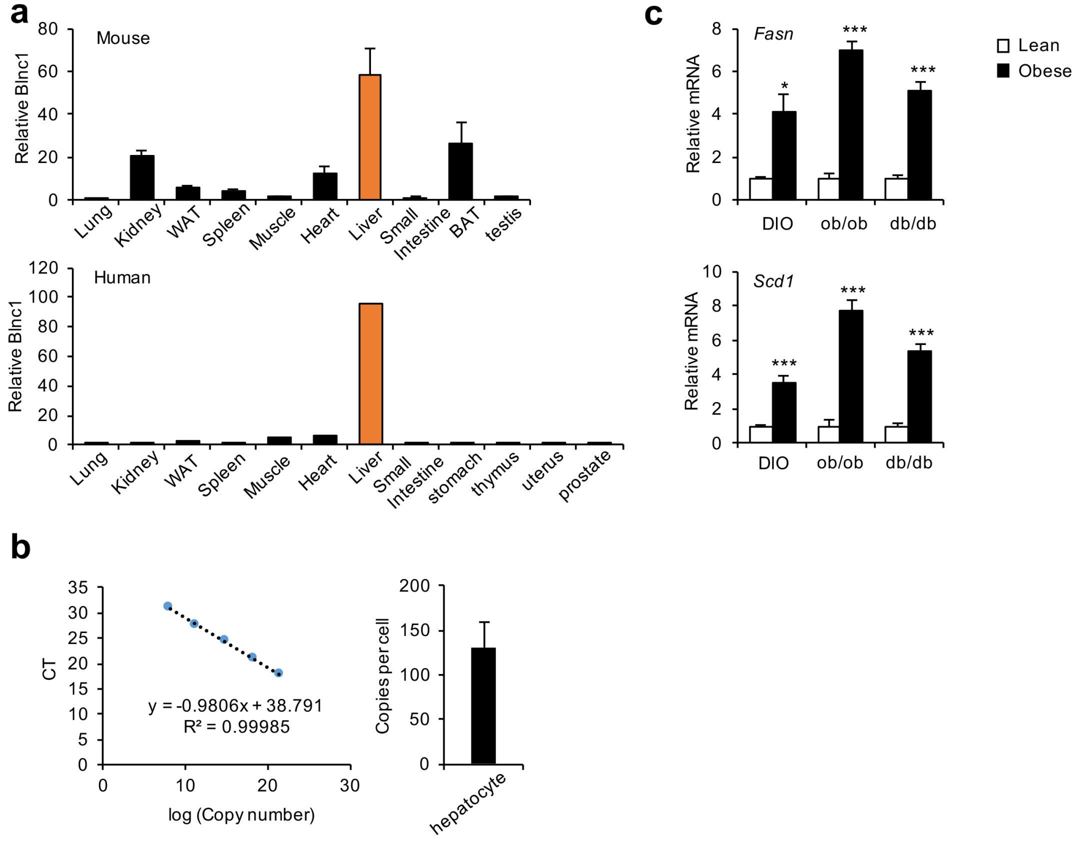

Supplementary Figure. 1. Blnc1 expression is enriched in the liver and linked to lipogenic induction in obesity. a qPCR analysis of Blnc1 expression in mouse and human tissues. The human tissue RNA panel was purchased from Clontech. **b** Copy number of Blnc1 RNA per hepatocyte (right). Standard curve generated using in vitro transcribed Blnc1 RNA (left). **c** qPCR analysis of Fasn and Scd1 mRNA expression in the livers from lean (open) and obese (filled) mice. For diet-induced obesity (DIO), WT mice were fed chow (n=5) or HFD (n=6) for 10 weeks. For genetic obesity, WT (n=5), ob/ob (n=3), or db/db (n=6) mice were fed standard chow. Data represent mean ± SEM. *p < 0.05, ***p < 0.001, obese vs. lean, two-tailed unpaired Student’s t-test.

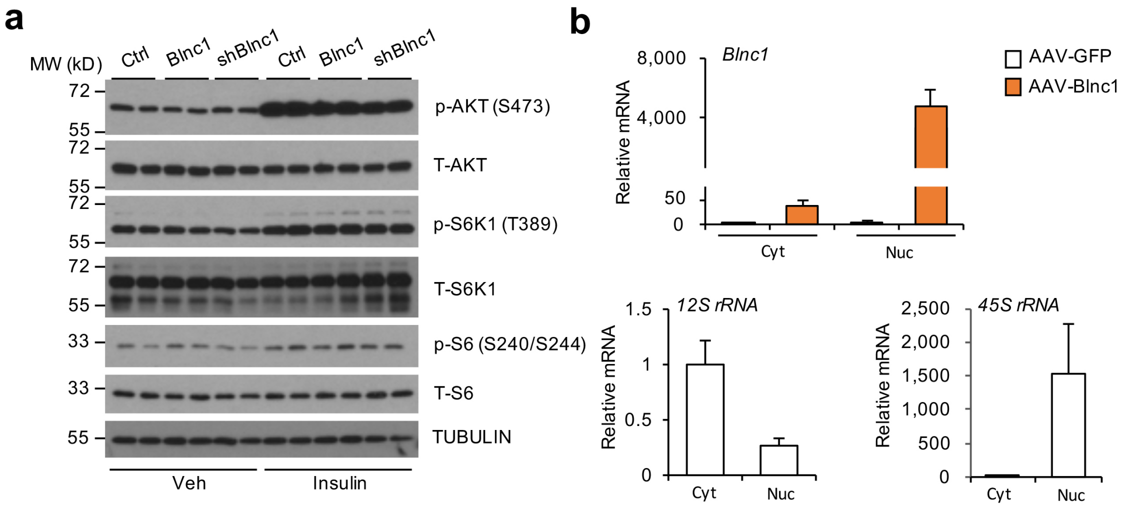

Supplementary Figure. 2. Subcellular localization of Blnc1 and its effects on insulin signaling. a Immunoblots of total lysates from primary hepatocytes transduced with GFP (Ctrl), Blnc1 or shBlnc1 adenovirus and treated with 100nM insulin for 15 min. **b** qPCR analyses Blnc1 expression in the cytosolic (Cyt) and nuclear (Nuc) fractions of mouse livers transduced with AAV-GFP (open, n=5) and AAV-Blnc1 (filled, n=7). Mitochondrial 12S rRNA and 45S rRNA were included as control for the cytosolic and nuclear fractions, respectively.

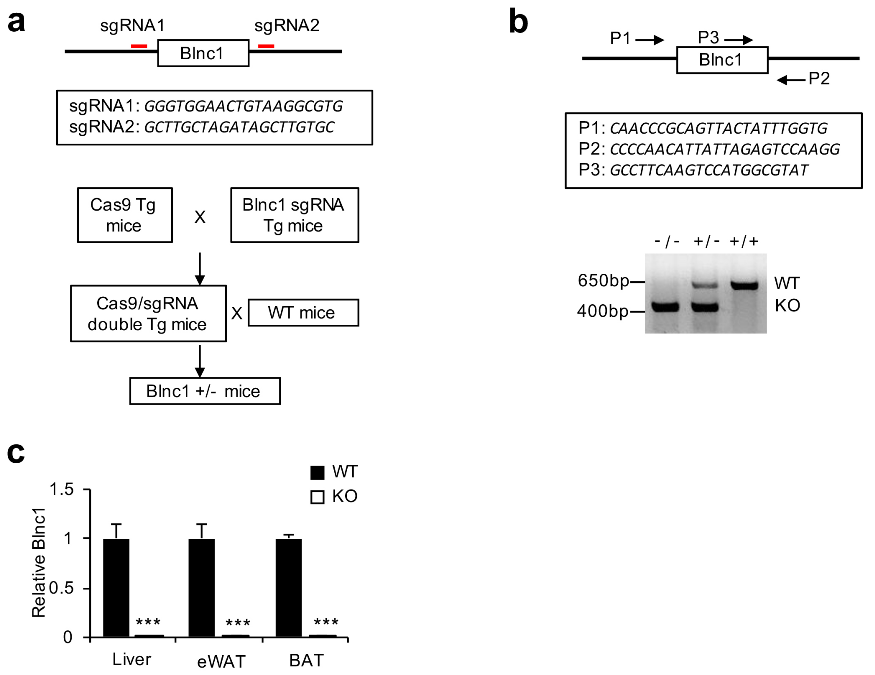

Supplementary Figure. 3. Generation of whole body Blnc1 knockout mice. a A schematic of the strategy for generating Blnc1 KO mice using CRISPR/Cas9. **b** Genotyping PCR. WT and KO bands were amplified using the indicated primers. **c** qPCR analysis of Blnc1 expression in WT (filled, n=5) and KO (open, n=5) mouse tissues. Data represent mean ± SEM. ***$p < 0.001$, WT vs. KO, two-tailed unpaired Student's t-test.

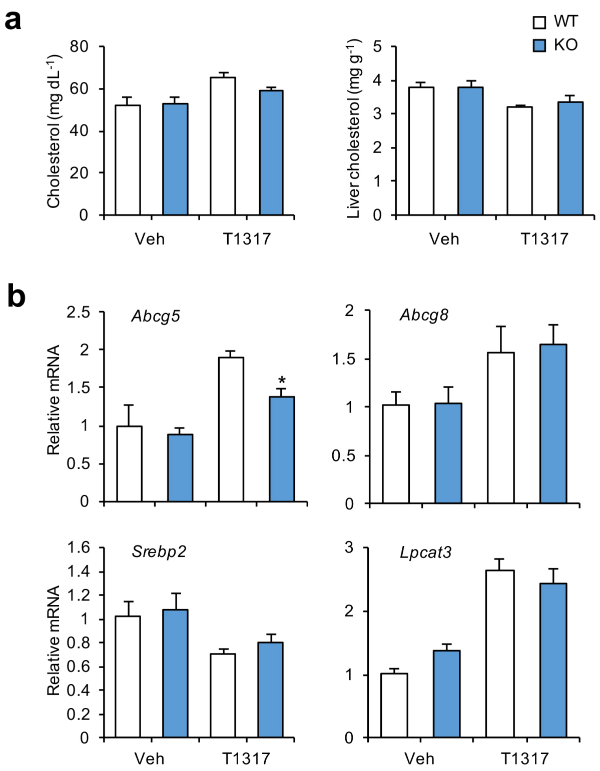

Supplementary Figure. 4 Effects of Blnc1 on cholesterol metabolism and hepatic gene expression. a Plasma and liver cholesterol content from WT (open) or Blnc1 KO (blue) mice receiving oral gavage of Veh (WT, n=4; KO, n=3) or T1317 (WT, n=3; KO, n=4) for 4 days. **b** qPCR analysis of hepatic gene expression in gavaged mice. Data represent mean ± SEM. *p < 0.05, WT vs. KO, two-tailed unpaired Student's t-test.

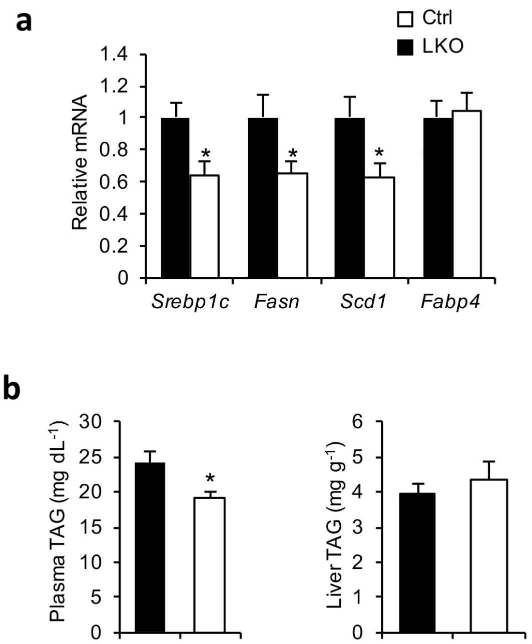

Supplementary Figure. 5. Blnc1 inactivation impairs lipogenic gene induction during refeeding. a qPCR analysis of hepatic gene expression in Ctrl (filled, n=9) or Blnc1 LKO (open, n=9) mice subjected to overnight fasting (16 hrs) followed by 8 hrs of refeeding. **b** Plasma and liver TAG content after refeeding. Data represent mean ± SEM. *$p < 0.05$, Ctrl vs. LKO, two-tailed unpaired Student's t-test.

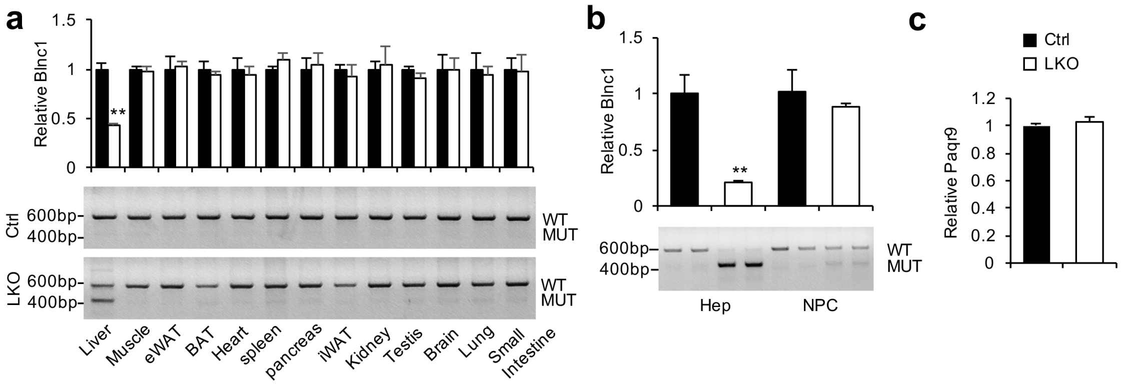

Supplementary Figure. 6. Liver-specific inactivation of Blnc1. a qPCR analysis of liver Blnc1 expression (top) and genotyping PCR (bottom) in tissues from Ctrl (filled, n=3) and Blnc1 LKO (open, n=3) mice. **b** qPCR analysis of Blnc1 expression in isolated hepatocytes (Hep) or non-parenchymal cells (NPC) from Ctrl (n=3) and LKO (n=3) mouse livers. **c** qPCR analysis of Paqr9 expression. Data in **b**-**c** represent mean ± SEM. **p < 0.01, Ctrl vs. LKO, two-tailed unpaired Student's t-test.

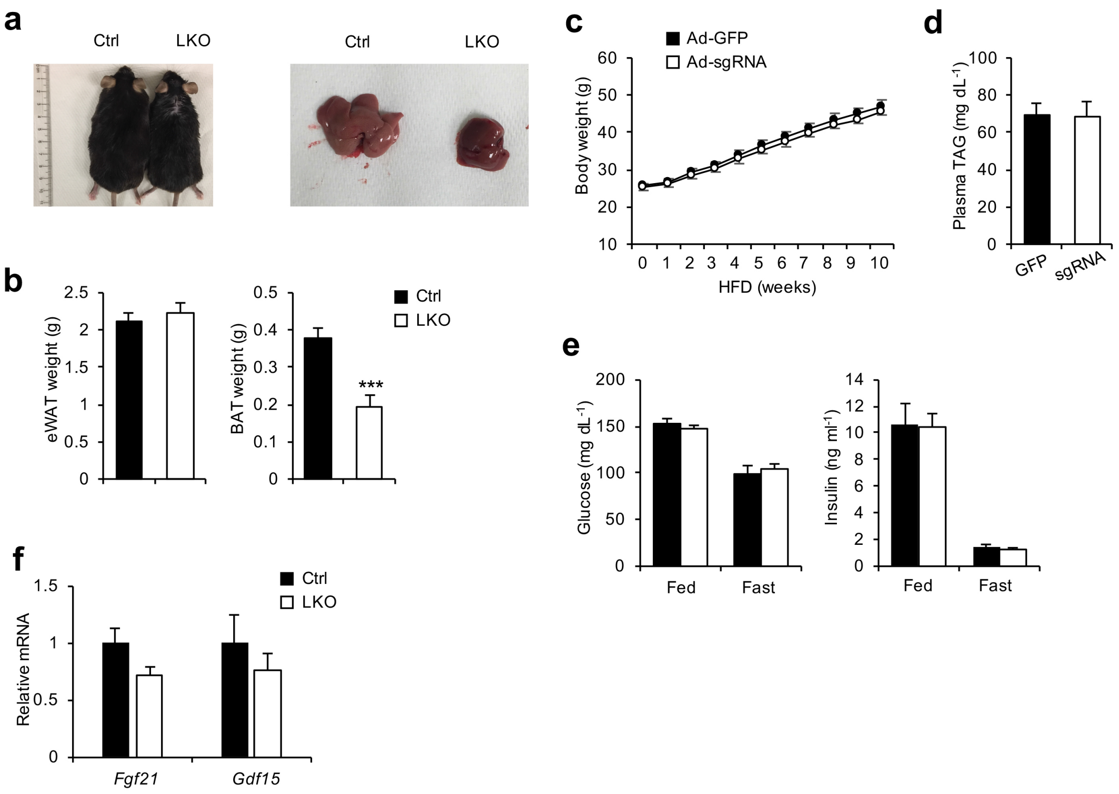

Supplementary Figure. 7. Hepatic expression of Blnc1 sgRNA does not alter metabolic parameters. a Appearance and liver morphology of Ctrl or LKO mice fed HFD for 10 weeks. **b** epididymal (eWAT) and brown fat (BAT) weight from Ctrl (filled, n=9) and LKO (open, n=9) mice fed HFD for 19 weeks. **c** Body weight of mice transduced with Ad-GFP (filled, n=6) or Ad-sgRNA (open, n=6) adenovirus and fed HFD for 10 weeks. **d** Plasma TAG levels. **e** Blood glucose (left) and plasma insulin (right) concentrations. **f** qPCR analysis of hepatic *Fgf21* and *Gdf15* expression in Ctrl (filled, n=9) and LKO (open, n=9) mice fed HFD for 19 weeks. Data in **b** represent mean ± SEM. ***$p < 0.001$, Ctrl vs. LKO, two-tailed unpaired Student's t-test.

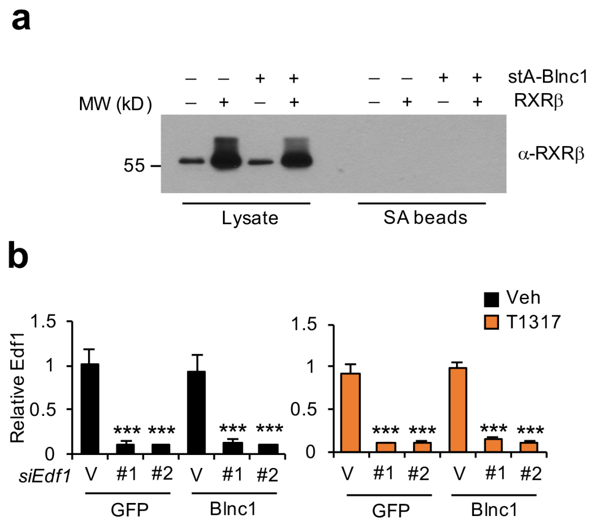

Supplementary Figure. 8. Physical interaction between Blnc1 and transcription factors. a Immunoblots of total lysates and precipitated proteins (SA beads) from HEK293T cells transiently transfected as indicated. **b** qPCR analysis of gene expression in transduced hepatocytes transfected with control siRNA (V) or siRNAs targeting EDF1 (#1 and #2) followed by treatments with Veh or 5μM T1317 for 24 hrs. Data represent mean ± SD (n=3). ***p < 0.001, Ctrl vs. #1 or #2, two-way ANOVA.

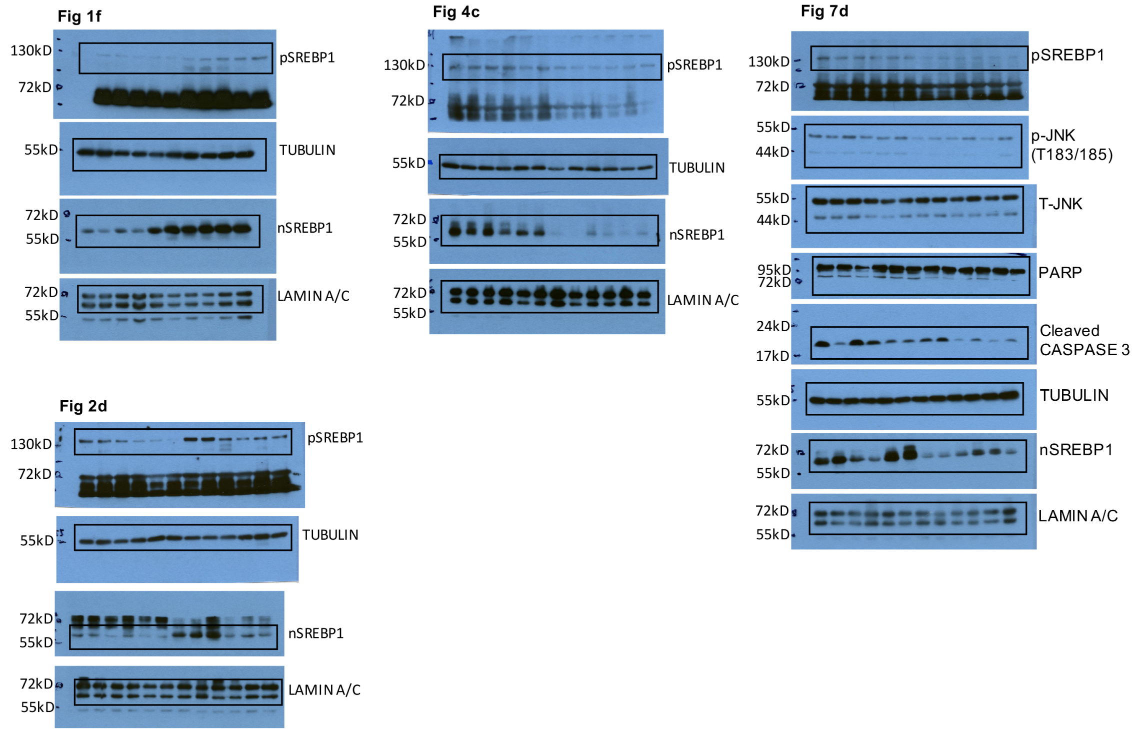

Supplementary Figure 9 Uncropped blots scans for Figure 1f, 2d, 4c and 7d.

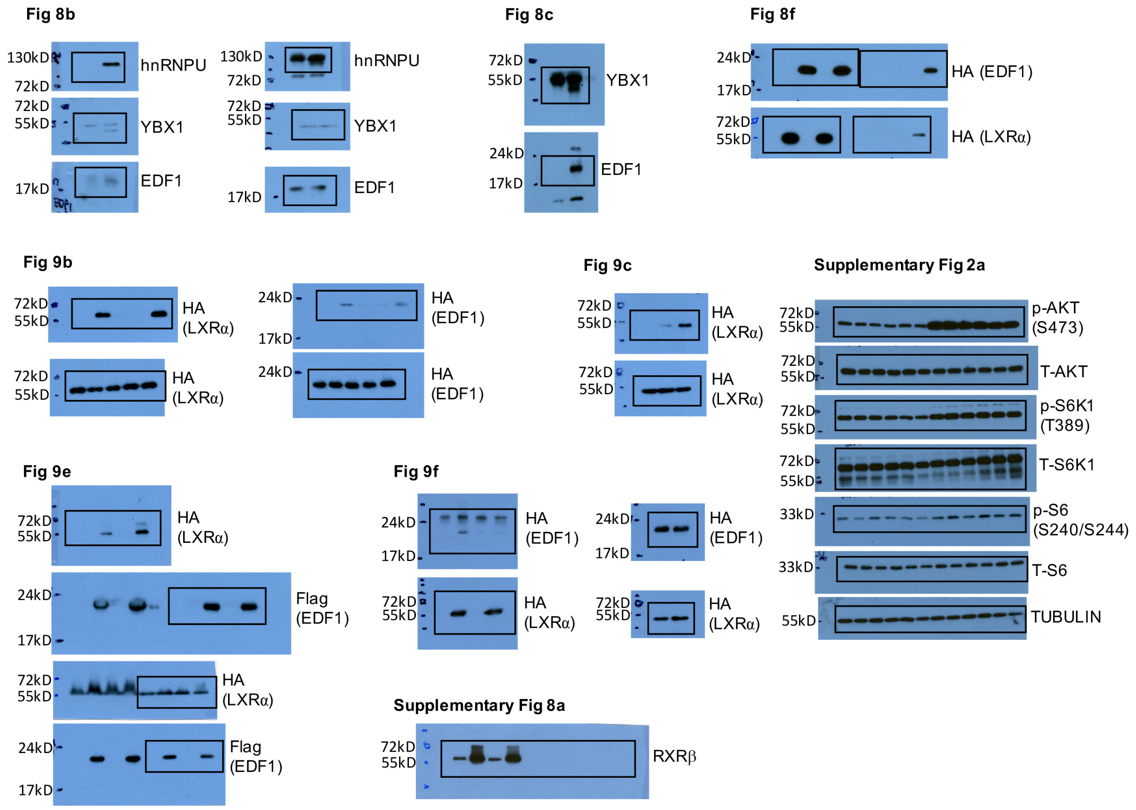

Supplementary Figure 10 Uncropped blots scans for Figure 8b-c, 8f, 9b-c, 9e-f and Supplementary Figure 2a and 8d.

Supplementary Fig 3b

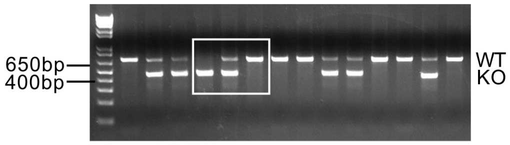

Supplementary Fig 6a

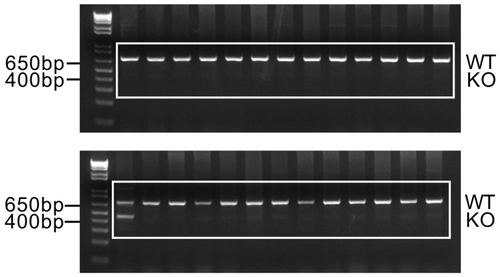

Supplementary Fig 6b

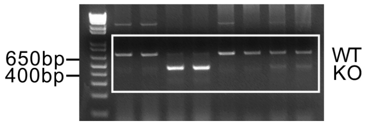

Supplementary Figure 11 Uncropped gels scans for Supplementary Figure 3b, 6a and 6b.

Supplementary Table 1. RNAseq expression values for hepatic genes in NASH-fed mice.

	WT-1	WT-2	WT-3	LKO-1	LKO-2	LKO-3		pvalue
Cyp17a1	3198	2179	2796	1722	950	1008	Down	3.0E-20
Acnat2	2037	1729	2419	1330	1164	981	Down	2.2E-08
Orm2	7851	10192	8221	6467	6637	4900	Down	3.2E-07
Slc3a1	2289	2389	2294	1804	1429	1509	Down	1.2E-06
Ccrn4l	4139	4078	4528	2516	2898	3584	Down	1.3E-06
Apoa4	586951	520148	509011	364798	369167	439268	Down	4.9E-06
Lcn2	16325	17679	12175	9593	13576	9961	Down	6.9E-06
Lyve1	2506	2019	2697	1340	1512	2050	Down	7.2E-06
Anxa2	16946	17169	15663	10565	14565	11387	Down	7.5E-06
Wfdc2	3096	2453	2734	1833	2137	1542	Down	8.1E-06
Srebf1	52837	43792	48788	34010	34234	40659	Down	1.3E-05
Abhd2	4007	4097	4761	2606	3534	3073	Down	1.4E-05
Acot2	2068	1788	2130	1242	1479	1529	Down	4.7E-05
Gbp2	1879	1802	2097	1352	1454	1440	Down	5.7E-05
Egr1	1671	2413	2219	1176	1692	1812	Down	6.9E-05
Jun	4306	4667	4511	2285	3333	3995	Down	1.0E-04
Col1a2	15299	12115	14751	9164	11636	9486	Down	1.2E-04
Bcl6	2574	2089	1477	1341	1692	1181	Down	1.4E-04
Slc16a5	3218	2740	3915	2530	2314	1922	Down	1.5E-04
Cxcl9	3695	3636	3764	3039	2916	2538	Down	1.5E-04
Igtp	4009	3746	4396	3247	3078	2976	Down	1.5E-04
Fgl1	33443	43549	27964	23287	28993	26252	Down	1.6E-04
Ccdc80	5154	4925	4986	3375	4443	3525	Down	1.6E-04
Col3a1	35709	28524	36093	23143	27848	21523	Down	1.7E-04
Mt1	7923	8223	7006	7065	6138	3822	Down	1.8E-04
Epha2	2530	2419	2307	1551	1805	1978	Down	1.8E-04
Frmd4b	7171	6389	7344	4863	5252	6151	Down	2.0E-04
Krt8	35631	37481	34188	23573	30674	29597	Down	2.1E-04
Col4a1	9800	10330	10599	7029	9490	6974	Down	2.2E-04
Ubd	3187	3519	2490	2256	3084	1774	Down	3.2E-04
Ppp1r3b	15045	14685	14265	10236	9475	13625	Down	3.3E-04
Serpinh1	4372	4651	4380	2979	3927	3585	Down	4.0E-04
Sdc1	14301	13814	15708	11775	12039	11174	Down	4.5E-04
Sall1	2036	1908	2015	1647	1539	1460	Down	7.1E-04
Aldh1b1	10403	6830	11262	8028	6583	6151	Down	8.5E-04
Col5a2	2168	1881	2335	1501	1759	1532	Down	1.0E-03
Cd93	2368	2477	2636	1823	2149	1949	Down	1.2E-03
H2-Q1	7890	6747	8055	4907	6139	6694	Down	1.6E-03
Stk38l	1738	1367	1663	1529	1046	1096	Down	1.7E-03
Tnfrsf12a	1723	1749	1475	983	1619	1078	Down	1.7E-03
Tfrc	3686	4175	5202	3801	2811	3081	Down	2.0E-03
Postn	3140	2497	2721	1930	2417	1983	Down	2.0E-03

Acsl3	2739	3362	3874	2621	2630	2201	Down	2.1E-03
Loxl2	1295	1244	1353	896	1124	952	Down	2.4E-03
Col5a1	2455	2206	2485	1613	2112	1791	Down	2.5E-03
Clca3a1	2186	2015	2272	1542	1821	1760	Down	2.8E-03
Synpo	2639	2488	2969	1796	2487	1907	Down	3.0E-03
Ccr2	2344	2185	2423	1620	1934	1994	Down	3.4E-03
Klf10	2345	3051	1920	1992	2177	1665	Down	3.5E-03
Col6a2	2795	2476	2466	1717	2383	1882	Down	3.6E-03
Mt2	3618	3891	3464	3513	3087	1794	Down	4.7E-03
Slc1a4	1172	1289	1351	989	1072	932	Down	4.8E-03
Sds	9560	7303	11554	9158	6302	6782	Down	5.6E-03
Cyr61	1561	1602	1175	1142	1191	1095	Down	7.1E-03
Ephb2	2372	2463	1734	1292	1821	1817	Down	7.5E-03
Acly	21301	20812	16167	13785	15194	17416	Down	9.6E-03
Ugt1a9	19356	5927	18166	6205	5217	10099	Down	1.6E-02
Tubb2a	13626	12745	10127	5584	8398	8185	Down	1.7E-02
Cebpd	1036	1268	1193	631	1139	1028	Down	1.7E-02
Cpxm1	2115	1906	2119	1499	2039	1343	Down	1.7E-02
Hk2	965	1459	1024	576	1205	968	Down	2.0E-02
Dusp1	2405	3026	1965	1523	2216	2166	Down	2.5E-02
Scand1	1883	1700	1582	947	1526	1553	Down	2.5E-02
Lgals3	5628	5052	5529	2831	4872	3159	Down	2.6E-02
Cntnap1	2902	3352	2198	1845	2315	2560	Down	3.1E-02
Col1a1	15541	11645	14716	8006	10928	8500	Down	3.1E-02
Gm6484	3069	3134	1210	1311	1125	1280	Down	3.8E-02
Hamp	14250	14791	13198	24524	23412	24545	Up	3.6E-19
Fbxo21	5930	5709	5295	8529	7124	7655	Up	5.9E-07
Nxpe2	2205	2117	2021	3086	2768	3353	Up	7.0E-07
Cyp3a11	185059	138054	225542	272482	216267	264795	Up	1.7E-06
Srd5a1	4596	4497	4893	7569	5065	7938	Up	5.3E-06
Nudt7	30016	33927	25673	48930	37380	44483	Up	5.8E-06
Npr2	3130	3416	2540	5205	3739	4437	Up	2.2E-05
Fcna	2549	2576	2252	3855	2964	3406	Up	3.4E-05
Cib3	1127	1494	986	1260	2641	1573	Up	3.9E-05
Cyp2e1	474764	469700	516727	744646	552886	643155	Up	4.5E-05
Mbd1	3064	3035	2929	3914	3702	4622	Up	4.5E-05
Ccbl1	4095	4073	4435	6442	4795	5773	Up	5.3E-05
Mup20	552351	786771	623800	835198	829270	1087553	Up	6.4E-05
Nkiras1	1906	1873	2177	2324	2416	3147	Up	1.3E-04
Ces2c	1684	1717	1678	3114	1889	2375	Up	1.7E-04
Clec4f	11585	8819	7087	14503	9864	13120	Up	3.5E-04
28104740	1906	2365	2247	3455	2790	2762	Up	3.7E-04
Wfdc17	1985	1997	1584	2606	2242	2641	Up	5.7E-04
Plcxd2	4479	5977	4610	7975	6154	6340	Up	7.6E-04

Cish	1071	2163	1203	2052	3186	1201	Up	8.1E-04
2010315I	1197	1249	1270	1930	1416	1618	Up	1.4E-03
Slc1a2	14601	13788	12318	21020	13744	18380	Up	1.4E-03
Ugt1a6b	1335	2381	1384	2690	2374	2087	Up	1.4E-03
Eif4ebp3	837	1470	1061	1377	1388	1648	Up	9.7E-03
Cyp2c44	9952	11402	13528	22546	15554	18075	Up	1.4E-02
Cyp4a12b	2311	3975	2540	8777	4562	12357	Up	1.7E-02
Cyp2c50	10716	11702	16725	30317	14715	22847	Up	3.2E-02
Gpcpd1	10316	11584	10521	20061	13000	15726	Up	3.3E-02
Sult2a8	8501	14896	9631	42300	16273	24110	Up	3.4E-02
Cyp4a12a	7720	9533	4316	19873	11601	31905	Up	3.7E-02
Elovl3	1544	2047	1455	3901	2026	3757	Up	3.7E-02
Gbp11	645	955	779	2287	1601	826	Up	4.9E-02

Supplementary Table 2: List of primer sequences.

Gene	5' Primer	3' Primer
**qPCR**		
Blnc1	CAAGGAAGTCATGAGCCCAATG	TAAAGGCTTCAACGGTGGCTG
Srebp1c	ATCGGCGCGGAAGCTGTCGG	GGGAAGTCACTGTCTTGGTTG
Fasn	GGTTACACTGTGCTAGGTGTTG	TCCAGGCGCATGAGGCTCAGC
Scd1	GCTGGAGTACGTCTGGAGGAA	TCCCGAAGAGGCAGGTGTAG
Fsp27	TCGACCTGTACAAGCTGAACCCT	AGGTGCCAAGCAGCATGTGACC
Ucp1	GGCATTCAGAGGCAAATCAGCT	CAATGAACACTGCCACACCTC
Cidea	GCAGCCTGCAGGAACTTATCAGC	GATCATGAAATGCGTGTTGTCC
Pparα	GCAGTGCCCTGAACATCGA	CGCCGAAAGAAGCCCTTAC
Ebf2	GGAACCGGAACGAGACCCCT	TCCCTTGGGTTTCCCGCTGT
Prdm16	CGGAAGAGCGTGAGTACAAATG	TCCGTGAACACCTTGACACAGT
Cox7a1	GTCTCCCAGGCTCTGGTCCG	CTGTACAGGACGTTGTCCATTC
Pgc-1α	AGCCGTGACCACTGACAACGAG	GCTGCATGGTTCTGAGTGCTAAG
Dio2	GATGCTCCCAATTCCAGTGT	TGAACCAAAGTTGACCACCA
Dgat2	TACTCCAAGCCCATCACCAC	CAGTTCACCTCCAGCACCTC
Gck	TCTGAGTGCATCTCTGACTTCC	CTTGTCTATGTCTTCGTGCC
Pfkl	AAGCCTATCTCATCCAGCTACG	CCCATCTTGCTACTCAGGATTC
ApoA4	ATCAAGAAGGAGCTGGAGGA	GGTGCTCCTGCAACTTCTGC
Fabp4	TGCCTTTGTGGGAACCTG	GCTTGTCACCATCTCGTTTTC
Pparγ	CCGTAGAAGCCGTGCAAGAG	GGAGGCCAGCATCGTGTAGA
Adgre1	ATCCTTGGCCATCCGGCAGA	GCAAAGCCAGGGTGGCAAGT
Ccr2	AAGAGGGCATTGGATTCACCACA	GCCGTGGATGAACTGAGGTAACA
Cx3cr1	GTTTTCCTGCAAGAATCGCAAG	CCTCAGGTCCCTCTTCATGTCA
Lcn2	GGCAATGCGGTCCAGAAAA	CCCTGGAGCTTGGAACAAATG
Mmp12	TGGAGCTCACGGAGACTTCAA	CAACAAGGAAGAGGTTTGTGCC
Mac2	CAGAGAGCACTACCCAGGAAAAT	TGAGGGTTTGGGTTTCCAGAG
Col1a1	AAGAGGCGAGAGAGGTTTCC	AGAACCATCAGCACCTTTGG
Col1a2	AGGTCCTAATGGAGATGCCG	CACAGGGCCTTCTTTACCAG

Acta2	CTGACAGAGGCACCACTGAA	CATCTCCAGAGTCCAGCACA
**ChIP**		
Srebp1c-ChIP	GACGCCGTCCGGATTCCGG	CGCCTTTAACCGCGTCTGCGC
18s-ChIP	AGTCCCTGCCCTTTGTACACA	CGATCCGAGGGCCTCACT
